# Exploration of Conditions for the Scaled Application of Laparoscopic Ovum Pick-Up in Sheep and Comparison of Follicular Development Differences Among Breeds

**DOI:** 10.3390/ijms26051989

**Published:** 2025-02-25

**Authors:** Dongxu Li, Xiangli Wu, Ying Chen, Yangsheng Wu, Gulimire Abudureyimu, Hongyang Liang, Xiuling Ma, Wei Zhang, Liqin Wang, Jiapeng Lin

**Affiliations:** 1 Biotechnology Research Institute, Xinjiang Academy of Animal Sciences, Urumqi 830000, China; 2022205013@stu.njau.edu.cn (D.L.); wuxiangli2022@163.com (X.W.); cy08272024@163.com (Y.C.); xj_wys@126.com (Y.W.); gulimire127@163.com (G.A.); 15701920082@163.com (H.L.); mxlde_123456@163.com (X.M.); zw02112024@163.com (W.Z.); 2 College of Animal Science and Technology, Nanjing Agricultural University, Nanjing 210095, China; 3 College of Animal Science and Technology, Xinjiang Agricultural University, Urumqi 830000, China

**Keywords:** sheep, aparoscopic ovum pick-up, transcriptomics, metabolomics, follicular development

## Abstract

In small ruminants, laparotomy for ovarian exploration followed by oocyte collection has been progressively replaced by laparoscopic puncture of follicles, which has become an important method for obtaining oocytes in vivo. However, the superovulation protocols and collection frequency used for laparoscopic ovum pick-up (LOPU) in sheep still require further investigation. This study explored the factors influencing LOPU efficiency in sheep, including Controlled Internal Drug Release (CIDR) for estrus synchronization, FSH source and dose, and recovery intervals. The optimal superovulation protocol (using the CIDR device, a total of 16 mg of long-acting recombinant ovine FSH (LR-FSH) administered in two doses, and a one-month interval between LOPU sessions) was subsequently identified. Ovarian follicles were collected via LOPU from Hu sheep and Altay sheep for transcriptomic and metabolomic sequencing to explore interbreed differences in follicular development. The results indicated that LOPU efficiency was significantly higher in the CIDR group (*p* < 0.05) and with a 30-day recovery interval (*p* < 0.05). No significant differences in LOPU efficiency were observed between FSH sources or hormone doses. Furthermore, Hu sheep exhibited significantly higher LOPU efficiency and more antral follicles than Altay sheep. Transcriptomic analysis of follicular contents and metabolomic profiling of follicular fluid revealed that differentially expressed genes and metabolites were primarily enriched in pathways related to steroidogenesis, amino acid metabolism, and fatty acid metabolism. This study provides an optimized treatment protocol to enhance LOPU efficiency and integrates multi-omics analyses to elucidate the molecular mechanisms underlying follicular development differences among various breeds.

## 1. Introduction

Altay sheep are renowned for their large body size and superior meat and fat production performance, making them one of Xinjiang’s premium local single-lamb sheep breeds, which have also been certified as a China National Geographical Indication Product [1]. In contrast, Hu sheep, a unique dual-purpose breed in China, valued for its meat, wool, and milk production, has significant economic value due to its year-round estrus and high reproductive capacity [2]. From a reproductive perspective, the fertility of Hu sheep far surpasses that of Altay sheep. Given these differences, investigating the follicular development mechanisms between single-lamb breeds like Altay sheep and multiple-lamb breeds like Hu sheep is essential for understanding the genetic and physiological basis of prolificacy in sheep. Such insights could provide a foundation for improving reproductive efficiency and optimizing breeding strategies in the industry.

Laparoscopic Ovum Pick-Up (LOPU) is a minimally invasive technique for retrieving live oocytes through follicular aspiration using a cannula under laparoscopic guidance [3]. This method offers the advantage of preserving the animal’s normal reproductive performance [4]. In modern livestock farming, laparoscopy is widely employed for various applications, including uterine horn insemination, embryo transfer, corpus luteum evaluation, ovulation monitoring, and early pregnancy diagnosis [5,6,7]. LOPU is typically performed after synchronization of estrus and superovulation treatments to obtain more high-quality follicles, thereby enhancing the quality of oocytes. Estrus synchronization is commonly achieved using Controlled Internal Drug Release (CIDR) devices, while superovulation is generally induced by injecting FSH (follicle-stimulating hormone) to stimulate ovarian follicle development. The combination of LOPU with in vitro fertilization (IVF) and embryo transfer technologies addresses several limitations of conventional in vitro embryo production and transfer methods, while significantly reducing the cost of producing high-quality embryos [8,9]. Therefore, the promotion and application of LOPU technology are crucial for the development of the livestock industry.

The primary factor limiting large-scale in vitro embryo production (IVEP) in small ruminants, such as sheep and goats, is the method of oocyte retrieval and its utilization rate [10]. While IVEP using oocytes from slaughterhouse-derived ovaries and frozen semen supports basic research, these oocytes often exhibit developmental asynchrony and unknown origins, resulting in low breeding value [11]. In contrast, LOPU for repeated oocyte collection from genetically superior females, ensures a supply of high-quality oocytes [12,13]. This significantly reduces the cost of producing high-quality embryos while maximizing the genetic potential of superior breeding females [14,15]. The integration of LOPU with IVEP technologies enhances the utilization and reproductive efficiency of high-genetic-value females, enabling the large-scale production of embryos and offspring with complete pedigrees and well-characterized genetic backgrounds [16]. This addresses the limited availability of high-quality embryos and accelerates the commercialization of embryo transfer. Furthermore, the application of LOPU in sheep increases selection intensity, shortens generation intervals, and fully capitalizes on the reproductive potential of superior genetic resources [17,18]. A robust IVEP system also provides a substantial supply of embryos for scientific research, including nuclear transfer, gene editing, and embryonic stem cells, while contributing to the conservation and reproduction of endangered species [19,20,21].

This study investigated the effects of hormone superovulation protocols, the use of CIDR, and recovery intervals on LOPU efficiency. Additionally, this study, through LOPU technology, found that the number of oocytes retrieved from Hu sheep was significantly higher than that from Altay sheep. By integrating transcriptomics and metabolomics analyses, we systematically explored the differences in follicle development. These findings provide a solid foundation for the large-scale implementation of LOPU technology in sheep reproduction and offer critical technical support for maximizing the reproductive potential of superior ewes.

## 2. Results

### 2.1. Effect of CIDR in Simultaneous Estrus on LOPU Efficiency in Altay Sheep

**Figure 1:**
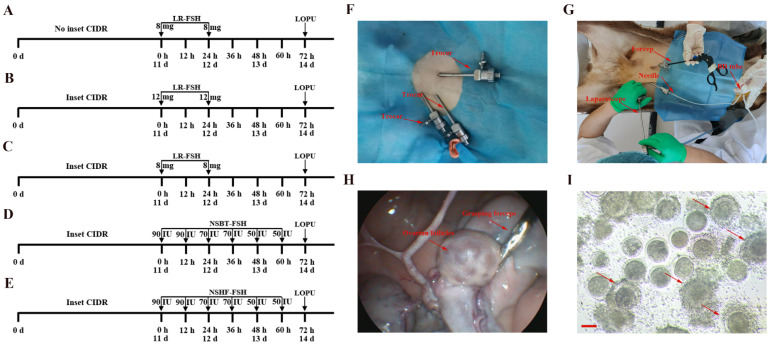
Experimental procedure of live ovum collection in sheep. (**A**–**E**) Procedure for simultaneous estrus and superovulation regimen. (**F**) The position of abdominal puncture, with the arrow pointing to the trocar. (**G**) The procedure showing three ports: the laparoscope, grasping forceps, and aspirating pipette/needle. (**H**) Laparoscope view showing the grasping forceps holding the ovarian base and ovarian follicle development. (**I**) Oocytes obtained via LOPU; the oocytes indicated by the arrow are used for in vitro fertilization. Scale bar, 100 µm.

The effects of CIDR in simultaneous estrus on LOPU efficiency were explored with reference to the trial protocols shown in Figure 1A,C, while maintaining consistent hormone treatments across both groups. As shown in Table 1, no significant differences were observed in the recovery rates of cumulus–oocyte complexes (COCs) between the CIDR-treated and the control groups (no CIDR). However, the CIDR-treated group exhibited significantly higher values regarding the average number of ovarian follicles, the average number of COCs, and the average number of viable COCs, compared to the non-CIDR group.

### 2.2. Effect of Different Hormone Doses in Superovulation Regimen on LOPU Efficiency in Altay Sheep

The effect of varying hormone doses on LOPU efficiency during superovulation was investigated according to the trial protocols shown in Figure 1B,C, while maintaining the same simultaneous estrus regimen. By comparing the 16 mg LR-FSH group with the 24 mg LR-FSH group in superovulation, there were no significant differences in the COCs recovery rate (%), the average number of ovarian follicles, the average number of COCs, and the average number of available COCs (Table 2).

### 2.3. Effects of Different Manufacturers of Hormones in Superovulation Regimen on LOPU Efficiency in Altay Sheep

The effects of hormones from different manufacturers on LOPU efficiency during superovulation were examined according to the trial protocols shown in Figure 1C–E, while maintaining the same simultaneous estrus regimen. As shown in Table 3, there were no significant differences observed in the COC recovery rate (%), the average number of ovarian follicles, the average number of COCs, and the average number of available COCs among the LR-FSH group, NSBT-FSH group, and NSHF-FSH group.

### 2.4. Effects of Interval Time on LOPU Efficiency in Altay Sheep

The effect of collection frequency on LOPU efficiency was explored by referring to the simultaneous estrus and superovulation regimen in Figure 1C. As shown in Table 4, the resulting outcomes of LOPU within 1 month showed that the COC recovery rate (%) was not affected by the frequency of oocyte collection. However, the average number of ovarian follicles, the average number of COCs, and the average number of available COCs in the first collection were significantly higher compared to those obtained after the 7-day and 15-day intervals, and there was no significant difference was observed between the first collection and the 30-day interval. Notably, the 7-day interval yielded the lowest LOPU efficiency.

### 2.5. Effects of Different Sheep Breeds on LOPU Efficiency

**Figure 2:**
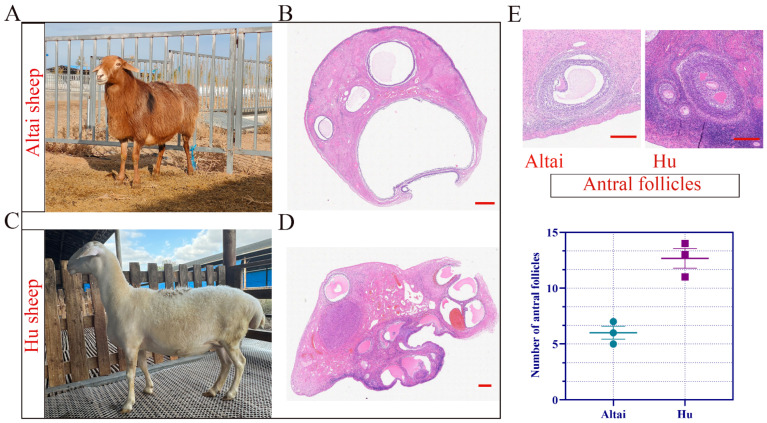
Comparison of ovarian follicle development between Hu sheep and Altay sheep. (**A**,**B**) HE staining of the largest transverse section of the Altai sheep ovary. Scale bar, 1 mm. (**C**,**D**) HE staining of the largest transverse section of Hu sheep ovaries. Scale bar, 1 mm. (**E**) The morphological characteristics of antral follicles and the number of antral follicles in Hu sheep and Altay sheep were statistically analyzed. Scale bar, 100 µm.

The effect of different sheep breeds on LOPU efficiency was explored by referring to the simultaneous estrus and superovulation regimen shown in Figure 1C. As shown in Table 5, there were no significant differences in COC recovery rates among the Hu sheep group, Fine wool sheep group, and Altay sheep group. However, the Hu sheep group exhibited a significantly higher average number of ovarian follicles, the average number of COCs, and the average number of available COCs compared to the Fine wool sheep and Altay sheep groups. In addition, as shown in Figure 2, histological analysis of the ovaries of Hu sheep and Altay sheep using HE staining revealed that the number of antral follicles in the ovaries of Hu sheep was significantly greater than that in Altay sheep.

### 2.6. Transcriptomic Sequencing Analysis of Follicular Contents from Hu Sheep and Altay Sheep

**Figure 3:**
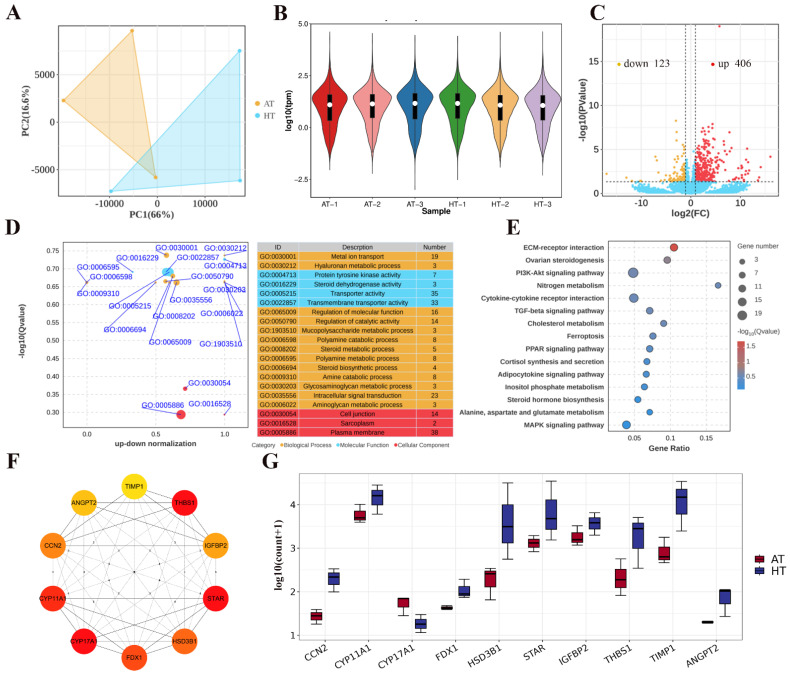
Transcriptome sequencing analysis. (**A**) PCA showing the genome-wide expression road map in follicular contents of Hu sheep and Altay sheep. (**B**) Violin plot of TPM in the follicular contents. (**C**) Volcano gene expression plot in Hu sheep and Altay sheep follicular contents. (**D**,**E**) GO and KEGG enrichment analysis of the DEGs. (**F**) Core composition in PPI protein interaction network map. (**G**) Bar chart showing the gene expression levels of the selected key genes.

As shown in Figure 3A, the PCA of the samples demonstrated reliable repeatability, confirming the robustness of the dataset. Gene expression distribution across different samples was visualized based on transcript per million (TPM) values, with expression distribution plots shown in Figure 3B. Differentially expressed genes (DEGs) were identified using a *t*-test with a significance threshold of *p* < 0.05. A volcano plot revealed a total of 529 DEGs, of which 406 were upregulated and 123 were downregulated (Figure 3C). To further investigate the biological functions of the DEGs, we performed GO and KEGG enrichment analyses. GO enrichment analysis indicated that the DEGs were primarily involved in processes such as the polyamine catabolic process, amine catabolic process, intracellular signal transduction, cell junction formation, and glycosaminoglycan metabolism (Figure 3D). KEGG pathway analysis revealed that the DEGs were significantly associated with several key pathways, including ECM-receptor interactions, ovarian steroidogenesis, the PI3K-Akt signaling pathway, the TGF-beta signaling pathway, cholesterol metabolism, inositol phosphate metabolism, and the MAPK signaling pathway (Figure 3E). Additionally, protein–protein interaction (PPI) network analysis was conducted for all differential genes. Using the MOED tool, we identified 10 critical genes related to steroidogenesis, including *THBS1*, *STAR*, *CYP11A1*, *CYP17A1*, and *FDX1* (Figure 3F,G). These genes are central to the regulation of ovarian function and steroid hormone synthesis.

### 2.7. Metabolomic Analysis of Follicular Fluid in Hu Sheep and Altay Sheep

**Figure 4:**
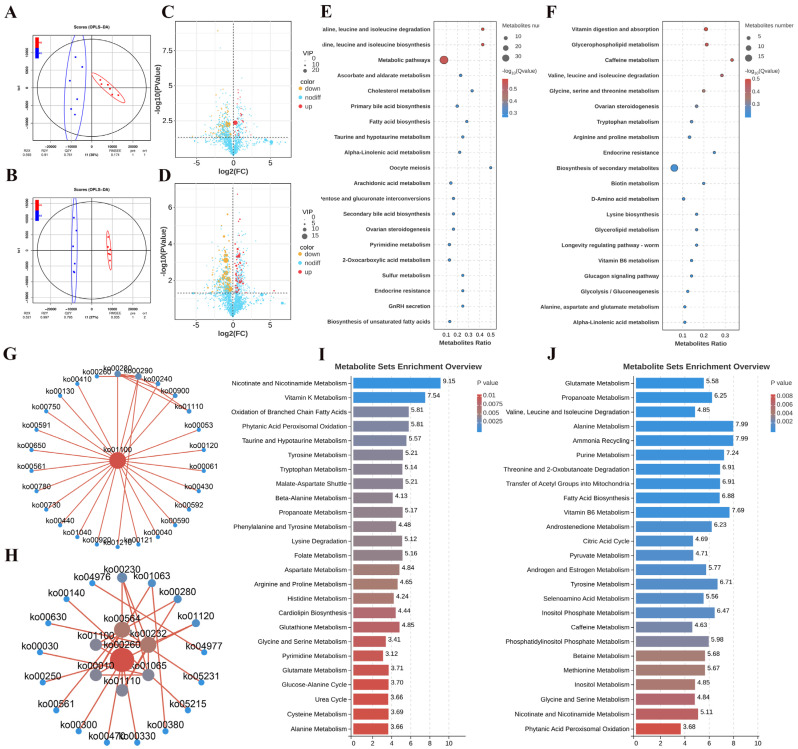
Differential metabolites and functional analysis. (**A**,**B**) OPLS-DA score scatter plot in NEG and POS. (**C**,**D**) Volcanic diagram of differential metabolites in NEG and POS. The red dots represent the differential metabolites with VIP ≥ 1 and *p* < 0.05, which are upregulated (FC > 1); the blue dots represent the differential metabolites with VIP ≥ 1 and *p* < 0.05, which are downregulated (FC < −1). The larger the dot, the greater the VIP value of the metabolite. (**E**,**F**) KEGG enrichment bubble chart in NEG and POS. The ordinate is the pathway, and the abscissa is the enrichment factor. (**G**,**H**) KEGG pathway interaction network diagram in NEG and POS. (**I**,**J**) MSEA enrichment map in NEG and POS. The ordinate indicates the name of the enriched metabolic set, and the abscissa indicates the enrichment degree.

As shown in Figure 4A,B, the OPLS-DA score scatter plots demonstrate a distinct separation between Hu sheep and Altay sheep. Differential metabolites were identified using a VIP score ≥ 1 and a *t*-test *p*-value < 0.05 as the screening criteria. In the negative ion mode (NEG), 119 differential metabolites were identified, including 23 upregulated and 96 downregulated (Figure 4C). In the positive ion mode (POS), a total of 169 differential metabolites were identified, with 68 upregulated and 101 downregulated (Figure 4D). KEGG pathway analysis was performed on the differential metabolites identified in both POS and NEG modes. In the NEG mode, the pathways involved included cholesterol metabolism, fatty acid biosynthesis, alpha-linolenic acid metabolism, and 2-oxocarboxylic acid metabolism (Figure 4E). In the POS mode, the affected metabolic pathways primarily included ovarian steroidogenesis, amino acid metabolism, glycerolipid metabolism, and alpha-linolenic acid metabolism (Figure 4F). Additionally, a pathway interaction network was constructed based on the enriched pathways from the differential metabolites. In the NEG mode, the metabolic pathways (Ko01100) were identified as the core pathways (Figure 4G). In the POS mode, core pathways included glycerophospholipid metabolism (Ko00564), glycolysis/gluconeogenesis (Ko00010), glycine, serine, and threonine metabolism (Ko00260), and caffeine metabolism (Ko00232) (Figure 4H). Metabolic Set Enrichment Analysis (MSEA) highlighted several amino acid metabolism-related and fatty acid metabolism pathways in NEG mode, such as tyrosine metabolism, tryptophan metabolism, and beta-alanine metabolism (Figure 4I). In the NEG mode, amino acid metabolism-related pathways, including glutamate metabolism, propanoate metabolism, alanine metabolism, and fatty acid biosynthesis, were significantly enriched (Figure 4J).

### 2.8. Association Analysis of Transcriptome and Metabolome Reveals the Molecular Mechanism of Follicle Development in Hu Sheep and Altay Sheep

**Figure 5:**
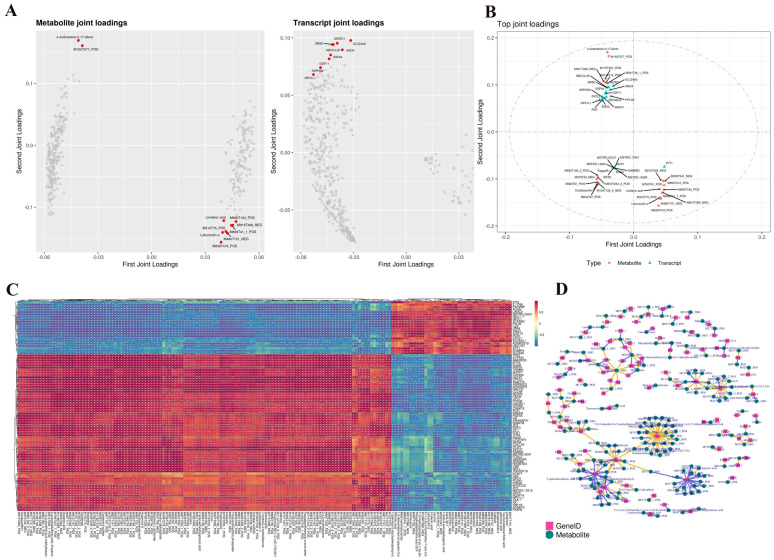
Metabolite and transcript joint loadings plots. (**A**) The O2PLS loadings plot of metabolites and transcripts. (**B**) The association loadings plots. The sign of the loading value indicates positive or negative correlation with another omics data type. The absolute value of the loading value indicates the strength of the correlation. (**C**) Heat map of correlation between gene expression and metabolite abundance. (**D**) Network diagram of the correlation between gene expression and metabolite abundance, where the yellow line indicates a positive correlation and the blue line indicates a negative correlation. * indicates a significant difference (*p* < 0.05).

The common pathway analysis revealed that both the DEGs and differential metabolites were enriched in 25 key pathways. Notably, we identified that ovarian steroidogenesis, biosynthesis of amino acids, and fatty acid biosynthesis pathways play significant roles in follicular development. Furthermore, the loadings plot and the loading diagram (Figure 5A) illustrate the relationships between metabolites and genes. In the metabolome, several metabolites were closely associated with genes, such as M287T278_POS (4-androstene-3,17-dione) and M277T45_NEG (linolenic acid). The transcriptome loading also revealed several genes related to metabolites, including *PATL2*, *MORC1*, and *GDF11* (Figure 5B). Additionally, a heatmap (Figure 5C) and a correlation network map (Figure 5D) were generated based on the top 250 differential genes and metabolites, with absolute correlation coefficients values of >0.5. These visualizations further highlight the strong correlations between specific genes and metabolites involved in follicular development.

## 3. Discussion

**Figure 6:**
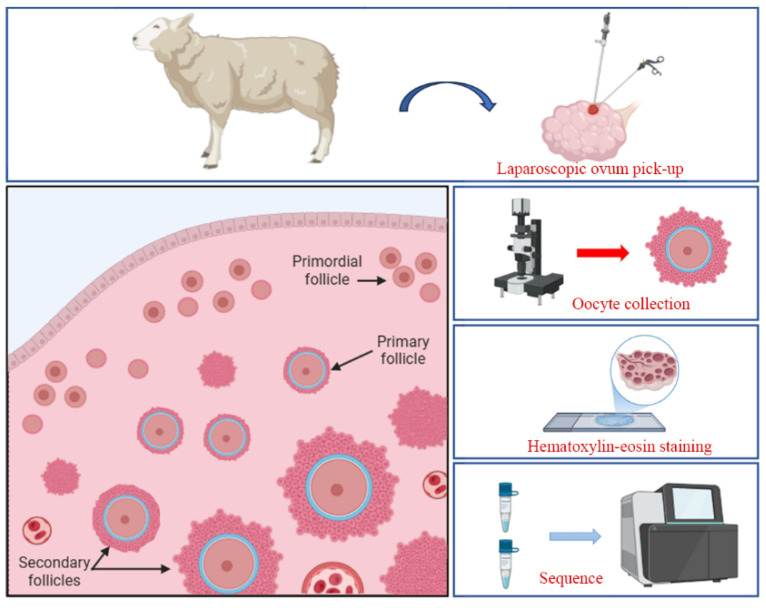
This study investigates the effects of hormone superovulation protocols, controlled internal drug release (CIDR) devices for estrus synchronization, and recovery intervals on the efficiency of laparoscopic ovum pick-up (LOPU) in sheep. Additionally, using the optimized LOPU method, this study explores differences in follicular development among sheep breeds through indicators such as oocyte collection, ovarian morphology, and omics analysis.

In recent decades, assisted reproductive technologies have advanced rapidly, encompassing estrus synchronization, artificial insemination (AI), multiple ovulation and embryo transfer (MOET), and IVEP. These technologies have become essential tools for expanding high-quality species, conserving endangered animals, and developing transgenic animals for medical purposes [22,23,24]. The livestock rapid propagation system, based on LOPU-IVEP, has significantly enhanced reproductive efficiency in female livestock, marking a major breakthrough in reproductive technology following the advent of artificial insemination. Particularly in cattle and sheep production, LOPU-IVEP technology addresses natural reproductive limitations, shortens generational intervals, and maximizes the genetic potential of elite female livestock [25,26]. Currently, LOPU has been successfully applied in animals unsuitable for transvaginal ultrasound-guided ovum pick-up, becoming the preferred method for retrieving oocytes from small ruminants [27,28]. This technique is minimally invasive, quick, repeatable, and does not impact the normal reproductive potential of the animals [29,30]. However, given the multiple factors affecting LOPU efficiency, it has yet to be widely applied in sheep production. Therefore, this study aimed to explore and optimize LOPU protocols in sheep, laying a solid foundation for the broader application of this technique in small ruminants (Figure 6).

This study explored the effects of various factors on the efficiency of LOPU in Altay sheep, including the use of CIDR during estrus synchronization, hormone dosages for superovulation, hormone sources, and ovum pick-up frequency. The results indicated that using CIDR for synchronization significantly improved both estrus rate and LOPU efficiency in sheep. Previous studies have suggested that the addition of progesterone during follicular development may enhance the developmental potential of oocytes [31]. Follicular development is a complex and dynamic process regulated by gonadotropins, which influence hormonal feedback in the hypothalamus and pituitary, thereby affecting follicular growth [32,33]. Follicular development progresses through both gonadotropin-independent and -dependent stages, with antral follicle growth dependent on FSH and LH [34,35]. The FSH dose and superovulation regimen from different manufacturers can impact follicle quality. In sheep production, the most commonly used and stable method for promoting follicle development remains multiple FSH injections [36]. Our findings revealed that neither the hormone source nor the dosage had a significant effect on LOPU yield. Some research indicates that although lower hormone doses may not affect the overall LOPU yield, they may improve oocyte quality [36,37]. Overall, the gonadotropin dose, stimulation protocols, and timing of administration are critical factors influencing both oocyte yield and quality. Additionally, optimizing the frequency of ovum pick-up is essential for maximizing the reproductive potential of high-quality females. In this study, the number of viable COCs obtained during the first LOPU was significantly higher than those collected at 7-day and 15-day intervals, with no significant difference observed between the first collection and the 30-day interval. This suggests that recovery time post-LOPU significantly influences oocyte yield. Previous studies have shown that while a 7-day interval may not negatively affect oocyte quality, it can lead to abdominal tissue damage [38]. To mitigate such adverse effects, a 30-day interval provides sufficient recovery time for ovarian function, effectively maintaining oocyte quantity and quality [39].

Based on preliminary research, we developed a protocol involving the use of CIDR for estrus synchronization and the administration of two doses of long-acting recombinant ovine FSH, totaling 16 mg, for superovulation. Follicular contents and follicular fluid were collected via LOPU to investigate the differences in follicular development among sheep breeds. In this study, we compared the morphological characteristics of ovarian follicles between Hu sheep and Altay sheep. Our statistical analysis revealed that the number of antral follicles after superovulation in Hu sheep was significantly higher than that in Altay sheep. Additionally, the average number of ovarian follicles, the average number of COCs, and the average number of viable COCs were all significantly greater in the Hu sheep group compared to the Altay sheep group. Based on these findings, we further explored the differences in follicular development between Hu and Altay sheep using multi-omics sequencing.

Transcriptome sequencing analysis revealed that DEGs were primarily enriched in pathways such as ovarian steroidogenesis, the PI3K-Akt signaling pathway, the TGF-beta signaling pathway, cholesterol metabolism, and inositol phosphate metabolism, among others. Ovarian steroidogenesis, which predominantly occurs within the follicle, is essential for normal reproductive function. Granulosa cells and theca cells (TCs) play pivotal roles in converting cholesterol into sex steroids, which are crucial for follicular development [40,41]. Steroidogenic activity increases as follicular development progresses, particularly during the late follicular phase, with preovulatory surges in progesterone (P4) and estradiol secretion [42]. Furthermore, proper ovarian steroidogenesis metabolism is crucial for follicular development and ovarian function [43]. The TGF-beta signaling pathway significantly regulates GC function and follicular development [44,45]. Our experimental results indicated that the expression levels of genes involved in ovarian steroidogenic pathways, such as *CYP11A1*, *STAR*, and *HSD3B1*, were significantly higher in Hu sheep compared to Altay sheep. CYP11A1 encodes an enzyme crucial for steroidogenesis, converting cholesterol to pregnenolone, thus initiating hormone synthesis [46]. STAR is involved in the transport of cholesterol across membranes, another key step in steroidogenesis [47]. HSD3B1 encodes 3β-hydroxysteroid dehydrogenase, which regulates steroid hormone biosynthesis and ovarian hormone secretion, influencing follicular development [48]. The differential expression of these genes may be one of the underlying reasons for the differences in follicular development between Hu and Altay sheep. In our study, higher expression of these genes in Hu sheep follicles may contribute to more follicular development and stronger ovarian function, promoting prolific reproductive characteristics. In contrast, the lower expression of these genes in Altay sheep may lead to fewer follicles and weaker ovarian function, thus explaining their single-lamb reproductive traits. These findings suggest that differences in ovarian steroidogenesis may be a key factor contributing to breed-specific variations in follicular development.

Metabolomic studies provide valuable insights into the dynamic changes in metabolites within follicles, offering a deeper understanding of the roles that various metabolic pathways play in follicular development. In this study, metabolomic analysis identified differential metabolites that were primarily enriched in pathways such as ovarian steroidogenesis, amino acid metabolism, glycerolipid metabolism, and alpha-linolenic acid metabolism. Amino acids that are crucial for follicular development, such as glutamine and aspartate, play key roles in cell metabolism, protein synthesis, and energy provision, all of which directly affect oocyte maturation [49]. The lipid droplets accumulate during oocyte growth, and fatty acid oxidation produces substantial amounts of ATP, which supports oocyte maturation and early embryonic development [50]. Studies have suggested that the amino acid and fatty acid composition in follicular fluid could serve as predictive indicators of in vitro embryonic development [51]. In the integrated transcriptomic and metabolomic analysis, we identified the differential gene *GDF11* and the metabolite M287T278_POS (4-androstene-3,17-dione) in the steroidogenesis pathway. Research indicates that *GDF11* downregulates the expression of steroidogenic regulatory proteins in human luteal granulosa cells via the ALK5-mediated SMAD3 signaling pathway [52]. Steroid hormones not only promote follicle growth and maturation but also play a decisive role in ovulation [53]. In our study, we observed differences in GDF11 expression between sheep breeds, which may contribute to breed-specific differences in ovulation. Through the above analysis, significant differences in steroidogenesis, as well as the essential amino acid and fatty acid content, were observed between the ovarian follicles of Hu sheep and Altay sheep. These differences may be the primary factors influencing the production of fully mature follicles.

In our analysis, we found a strong correlation between the changes in certain metabolites and the expression patterns of related genes. For instance, genes involved in steroidogenesis (such as CYP11A1, STAR) showed consistent changes with specific fatty acid metabolites, suggesting that these metabolites might be jointly regulated with gene expression during follicular development. Additionally, the expression of genes related to amino acid metabolism showed a high correlation with changes in amino acid metabolites, further validating the impact of gene regulation on metabolic processes during follicle development. However, we also observed some discrepancies between gene expression and metabolite changes. In certain metabolic pathways, although related genes showed significant changes, the corresponding metabolites showed only minor variations. This could indicate that other factors are involved in regulating these pathways, or there may be complex feedback mechanisms. This study combines transcriptomics and metabolomics to reveal the differences in gene expression and metabolite changes during follicular development in Hu sheep and Altay sheep, providing new insights into the molecular mechanisms of follicular development. It helps identify key genes and metabolic pathways affecting reproductive capacity, offering theoretical support for improving sheep breeds and enhancing reproductive efficiency, with significant scientific and practical implications.

## 4. Material and Methods

### 4.1. Animals

The experimental animals were Altai sheep from Fuluyuan Sheep Farm (Altai, China), Fine wool sheep from Changji Kangpson Sheep Farm (Altai, China), and Hu sheep from Shangpin Sheep Company (Changji, China). The experiment selected a total of 208 adult sheep of three breeds, aged 2 to 3 years, with a body weight ranging from 45 to 55 kg, all of which were participating in superovulation trials for the first time. The reproductive health of all animals was confirmed through ultrasonographic examination, ensuring the absence of purulent or mucoid vaginal secretions, clinical or subclinical endometritis, reproductive system diseases, microbial contamination, or ovarian cysts. All animals were managed under semi-intensive farming systems, housed indoors day and night, and fed a diet comprising roughage, silage, and concentrates.

### 4.2. Hormones

The Controlled Internal Drug Release (CIDR; Pharmacia and Upjohn, Hartwell, Australia) is inserted into ewes for 10 days to synchronize estrus, followed by superovulation treatment using follicle-stimulating hormones (FSH) from different manufacturers. FSH from Ningbo SanSheng Biological Technology (NSBT-FSH, Ningbo, China) and Ningbo Second Hormone Factory (NSHF-FSH, Ningbo, China) are derived from porcine pituitary, whereas long-acting recombinant ovine FSH (LR-FSH; Youliankang Pharmaceutical Technology Co., Ltd., Guangzhou, China) is produced through genetic recombination technology. This process involves expression in CHO cells, followed by glycosylation and PEGylation, yielding a high-activity FSH protein. Compared to traditional FSH, long-acting recombinant FSH presents several advantages: (1) Efficient large-scale production; (2) Enhanced purity and consistency; (3) Extended half-life, reducing the frequency of injections; (4) Reduced immunogenicity, minimizing the risk of allergic reactions. These characteristics render long-acting recombinant FSH advantageous for clinical applications, especially in superovulation and estrus synchronization protocols.

### 4.3. Experimental Design

In experiment 1, estrus synchronization was performed in all animals using CIDR, in addition to the protocol shown in Figure 1A. Group A animals (Figure 1A) received LR-FSH 8 mg each, administered 24 h apart, for superovulation. Group B animals (Figure 1B) were treated with two injections of 12 mg LR-FSH, given 24 h apart, for superovulation. The superovulation protocol for Group C animals was identical to that of Group A (Figure 1C). Group D animals (Figure 1D) received 420 IU of NSBT-FSH, administered twice daily in decreasing doses over 3 days (90/90, 70/70, and 50/50 IU). Group E animals (Figure 1E) were treated with 420 IU of NSHF-FSH, administered twice daily in a similar decreasing dose regimen over 3 days (90/90, 70/70, and 50/50 IU). Laparoscopic ovum pick-up (LOPU) was performed 72 h after treatment. The recovery rate of cumulus-oocyte complexes (COCs), as well as the average number of ovarian follicles, the average number of COCs, and the average number of available COCs, were recorded.

In experiment 2, Hu sheep and Altai sheep were treated according to the regimen outlined in Figure 1C, with estrus synchronization achieved using CIDR and two injections of LR-FSH (8 mg each), administered 24 h apart (*n* = 13), for superovulation. Upon estrus detection, Follicle fluid collection was performed via LOPU from follicles larger than 5 mm in diameter, and centrifuged at 1500 rpm for 5 min. The supernatant was then collected for metabolomic sequencing, while the precipitated material was used for transcriptomic analysis. Additionally, the ovaries were surgically harvested for histological examination.

### 4.4. Laparoscopic Ovum Pick-Up (LOPU)

The LOPU procedure, based on previous studies [54], can be summarized as follows. After sterilizing the area along the midline of the udder, the sheep is anesthetized and securely positioned. A laparoscope (Bewick Biotechnology LLC, Beijing, China) is then used to locate the uterus and ovaries following puncture. After stabilizing the ovary, follicular fluid is aspirated (Figure 1F–H). The retrieved oocytes are transferred into 14 mL BD tubes (BD Falcon, Corning, NY, USA) containing maturation medium. In a sterile laboratory, the oocytes are carefully selected under a stereomicroscope (SMZ-645; Nikon, Tokyo, Japan), washed multiple times, and subsequently cultured in a maturation medium.

### 4.5. Grading of Oocyte Quality

Oocytes were classified into 4 grades according to the morphological characteristics of the COCs. Grade A: homogeneous cytoplasm, encapsulated by at least three layers of granulosa cells; Grade B: homogeneous cytoplasm, encapsulated by 1–2 layers of granulosa cells; Grade C: homogeneous cytoplasm with no granulosa cells, classified as naked oocytes; Grade D: inhomogeneous or semi-transparent cytoplasm, indicative of dead or degenerated oocytes. Grades A and B oocytes were considered viable and suitable for subsequent culture (Figure 1I).

### 4.6. Histological Studies

Hematoxylin and eosin (HE) staining was performed to observe the morphological characteristics of follicular tissues in sheep ovaries. Freshly excised ovarian tissue was fixed in 4% paraformaldehyde (Biosharp, Hefei, China) for 48 h, and then sectioned along the largest transverse plane. The tissue was subsequently dehydrated through a series of graded alcohol concentrations and cleared in xylene. The cleared tissue block was embedded in melted paraffin and sectioned into 5-μm thick slices using a microtome (FPHIS-ARIES, Shanghai, China). The sections were floated in 40 °C water to flatten, adhered to glass slides, and then dried in a 45 °C incubator. Following this, HE staining was performed, after which the sections were deparaffinized with dimethylbenzene xylene. Finally, the slides were mounted for microscopic examination. The stained sections were observed under a light microscope (Nikon, Tokyo, Japan) for histological analysis.

### 4.7. RNA Library Construction and Sequencing

Total RNA was isolated and purified from the samples (3 replicates per group) using the TRIzol reagent. The quantity and purity of the RNA were assessed using a NanoDrop ND-1000 spectrophotometer (NanoDrop, Wilmington, DE, USA), and RNA integrity was evaluated using a Bioanalyzer 2100 (Agilent, Palo Alto, CA, USA). Polyadenylated (PolyA) mRNA was selectively captured using oligo(dT) magnetic beads (Dynabeads Oligo(dT), Thermo Fisher, cat. 25-61005, Waltham, MA, USA) through two rounds of purification. The captured mRNA was then fragmented under high-temperature conditions using the NEBNext^®^ Magnesium RNA Fragmentation Module (NEB, cat. E6150S, Ipswich, MA, USA). The fragmented RNA was reverse transcribed into complementary DNA (cDNA) using Invitrogen SuperScript™ II Reverse Transcriptase (cat. 1896649, Palo Alto, CA, USA). Subsequently, double-stranded cDNA synthesis was carried out using E. coli DNA polymerase I (NEB, cat. M0209, Ipswich, MA, USA) and RNase H (NEB, cat. M0297, Ipswich, MA, USA), converting the DNA–RNA hybrid into double-stranded DNA, which was then size-selected and purified using magnetic beads. Next, the double-stranded cDNA was treated with UDG enzyme (NEB, cat. M0280, MA, Ipswich, MA, USA) to remove the uracil-containing strand, and strand-specific libraries were generated by PCR, with an average fragment size of 300 bp ± 50 bp. Finally, paired-end sequencing (PE150) was performed using the Illumina NovaSeq™ 6000 system (LC Bio-Technology Co., Ltd., Hangzhou, China), following standard protocols.

### 4.8. Ultra-High Performance Liquid Chromatography-Electrospray Tandem Mass Spectrometry (UHPLC-MS/MS) Analysis

The samples were thawed at 4 °C, and 100 μL aliquots were mixed with 400 μL of cold methanol/acetonitrile (1:1, *v*/*v*) to precipitate proteins. The supernatant was then dried using a vacuum centrifuge. For liquid chromatography–mass spectrometry (LC-MS) analysis, the dried samples were re-dissolved in 100 μL of a 1:1 (*v*/*v*) acetonitrile/water solution. Chromatographic separation was performed using an ultra-high-performance liquid chromatography (UHPLC) system (1290 Infinity LC, Agilent Technologies, Santa Clara, CA, USA) coupled to a quadrupole time-of-flight mass spectrometer (AB Sciex TripleTOF 6600, Shanghai, China). Hydrophilic interaction liquid chromatography (HILIC) separation was carried out on a 2.1 mm × 100 mm ACQUITY UPLC BEH 1.7 μm column (Waters, Ireland). The raw mass spectrometry (MS) data were converted into MzXML files using the ProteoWizard MSConvert tool (v3.0.6428) and subsequently imported into XCMS software (online 3.7.1) for further processing. To identify differential metabolites among the comparison groups, orthogonal partial least squares discriminant analysis (OPLS-DA) was performed. The variable importance in projection (VIP) score derived from the OPLS-DA model, combined with the *p*-value from the univariate *t*-test, was used to screen for significant metabolites. The thresholds for differential metabolites were set as VIP ≥ 1 and *t*-test *p* < 0.05.

### 4.9. Functional Annotation of Differentially Expressed Genes (DEGs)

Transcriptomic and metabolomic integrated analysis is based on the individual standard analysis results of the two omics, linking the annotated results of differentially expressed genes and metabolites within metabolic pathways for a comprehensive analysis. Enrichment analysis and functional annotation of the differentially expressed genes (DEGs) were performed using the Kyoto Encyclopedia of Genes and Genomes (KEGG; www.genome.jp/kegg; accessed on 10 October 2024) and Gene Ontology (GO; http://geneontology.org; accessed on 10 October 2024) databases. GO and KEGG terms with *p*-values < 0.05 were identified through functional annotation of DEGs using the DAVID bioinformatics resources. Additionally, Gene Set Enrichment Analysis (GSEA) was conducted using GSEA software (version 3.10.1) and other available resources within the software suite.

### 4.10. Statistical Analysis

The normality of the data distribution was assessed using the Kolmogorov–Smirnov goodness-of-fit test. Statistical comparisons were performed using one-way analysis of variance (ANOVA) followed by Duncan’s or Tukey’s post-hoc tests. All statistical analyses were conducted using SPSS v27.0 (Chicago, IL, USA). Data are presented as the mean ± standard error (SE), and differences were considered statistically significant at *p* < 0.05.

## 5. Conclusions

This study demonstrates that estrus synchronization using the CIDR device, combined with two doses of long-acting recombinant ovine FSH (total 16 mg) for superovulation, followed by a one-month interval between LOPU sessions, is both effective and feasible for performing LOPU in sheep. Notably, the number of antral follicles post-superovulation was significantly higher in Hu sheep compared to Altay sheep. Additionally, significant differences in steroidogenesis, as well as in the content of essential amino acids and fatty acids, were observed between the ovarian follicles of the two breeds. This study aims to optimize the large-scale application of LOPU technology in sheep, thereby enhancing reproductive efficiency and promoting the genetic improvement of sheep breeds. Future research could explore the variability in LOPU outcomes across individual sheep or different breeds, as well as identify any technical challenges that could be addressed to further enhance the procedure.

## Figures and Tables

**Table 1:** Effect of CIDR on LOPU efficiency.

Group	No. of Donors	COCs Recovery Rate (%)	No. of Average Ovarian Follicles	No. of Average COCs	No. of Average Available COCs
Inset CIDR	15	95.65 ± 3.33	14.20 ± 0.92 ^a^	14.13 ± 0.93 ^a^	12.73 ± 0.83 ^a^
No inset CIDR	11	99.58 ± 2.40	9.64 ± 0.51 ^b^	9.09 ± 0.53 ^b^	7.82 ± 0.50 ^b^

Note: ^a,b^ values with different superscript letters within individual columns are statistically different (*p* < 0.05). CIDR: controlled internal drug release.

**Table 2:** Effect of hormone dose on LOPU efficiency.

Group	No. of Donors	COCs Recovery Rate (%)	No. of Average Ovarian Follicles	No. of Average COCs	No. of Average Available COCs
16 mg LR-FSH	13	100.33 ± 2.72	13.38 ± 0.75	14.46 ± 0.85	12.85 ± 0.85
24 mg LR-FSH	13	97.81 ± 2.38	14.23 ± 0.64	13.92 ± 0.72	12.38 ± 0.58

Note: LR-FSH: long-acting recombinant ovine FSH.

**Table 3:** Effect of different manufacturers’ hormones on LOPU efficiency.

Group	No. of Donors	COCs Recovery Rate (%)	No. of Average Ovarian Follicles	No. of Average COCs	No. of Average Available COCs
LR-FSH	24	97.58 ± 1.80	14.63 ± 0.60	14.21 ± 0.56	13.33 ± 0.54
NSBT-FSH	14	98.47 ± 3.64	13.14 ± 0.92	13.00 ± 1.07	12.21 ± 0.96
NSHF-FSH	12	99.46 ± 2.61	15.33 ± 0.98	15.17 ± 0.99	12.92 ± 0.69

Note: LR-FSH: long-acting recombinant ovine FSH; NSBT-FSH: Ningbo Sansheng Biological Technology Co., Ltd. (Ningbo, China) FSH; NSHF-FSH: Ningbo Second Hormone Factory Co., Ltd. (Ningbo, China) FSH.

**Table 4:** Effect of interval time on LOPU efficiency.

Group	No. of Donors	COCs Recovery Rate (%)	No. of Average Ovarian Follicles	No. of Average COCs	No. of Average Available COCs
0-day interval	12	99.52 ± 2.78	15.75 ± 0.86 ^a^	15.67 ± 0.89 ^a^	14.33 ± 1.02 ^a^
7-day interval	10	93.96 ± 4.43	8.50 ± 0.52 ^b^	7.60 ± 0.52 ^b^	6.50 ± 0.52 ^b^
15-day interval	12	93.57 ± 7.16	12.75 ± 0.94 ^cd^	11.58 ± 0.88 ^cd^	10.50 ± 0.91 ^cd^
30-day interval	12	99.27 ± 6.56	13.50 ± 0.95 ^ad^	13.33 ± 1.25 ^ad^	12.25 ± 1.17 ^ad^

Note: ^a–d^ values with different superscript letters within individual columns are statistically different (*p* < 0.05). 0-day interval: indicates the first LOPU in a one-month cycle; 7-day, 15-day, and 30-day intervals: indicate the second, third, and fourth LOPU in a one-month cycle (intervals 7, 15, and 30 days from the first time).

**Table 5:** Effects of different sheep breeds on LOPU efficiency.

Group	No. of Donors	COCs Recovery Rate (%)	No. of Average Ovarian Follicles	No. of Average COCs	No. of Average Available COCs
Hu sheep	18	95.77 ± 1.29	23.72 ± 1.36 ^a^	22.78 ± 1.41 ^a^	21.72 ± 1.39 ^a^
Fine wool sheep	18	96.39 ± 1.60	14.56 ± 0.77 ^b^	14.00 ± 0.76 ^b^	13.00 ± 0.79 ^b^
Altay sheep	24	96.52 ± 2.12	12.96 ± 0.52 ^b^	12.54 ± 0.59 ^b^	11.75 ± 0.57 ^b^

Note: ^a,b^ values with different superscript letters within individual columns are statistically different (*p* < 0.05).

## Data Availability

The original contributions presented in this study are included in the article. Further inquiries can be directed to the corresponding authors.
